# Harnessing the multidimensional bioactivity of *Chaetomorpha aerea*: Integrative phytochemical profiling with in vitro, in vivo, and in silico insights

**DOI:** 10.1002/ame2.70064

**Published:** 2025-07-11

**Authors:** Md. Mahmudul Hasan, Md. Abdul Alim, Md. Safayat Hossen Momen, Md. Shahidul Islam, Sajjad Hossen Chowdhury, Mohammad Rashed, Fahmina Hoque, S. M. Moazzem Hossen

**Affiliations:** ^1^ Department of Pharmacy, Faculty of Biological Sciences University of Chittagong Chittagong Bangladesh; ^2^ Department of Applied Chemistry and Chemical Technology University of Science and Technology Chittagong Chittagong Bangladesh; ^3^ Department of Pharmacy, Faculty of Basic Medicine and Pharmaceutical Technology University of Science and Technology Chittagong Chittagong Bangladesh; ^4^ Department of Pharmacy State University of Bangladesh Dhaka Bangladesh; ^5^ Department of Biotechnology and Genetic Engineering University of Development Alternative Dhaka Bangladesh

**Keywords:** anthelmintic, antiarthritic, anti‐inflammatory, antioxidant, *Chaetomorpha aerea*, computational chemistry, seaweed, α‐amylase

## Abstract

**Background:**

*Chaetomorpha aerea*, a marine green alga, has drawn attention because of its rich phytochemical constituents and therapeutic benefits. Using an integrated approach that combined in vitro, in vivo, and in silico approaches, this work examined the antioxidant, anti‐inflammatory, and antidiabetic qualities of acetone extract of *C. aerea* (AECA).

**Methods:**

Total phenolic and flavonoid concentrations of AECA were measured. Antioxidant activity was assessed using the DPPH and ABTS free radical scavenging assays. In vitro protein denaturation and in vivo carrageenan‐induced paw edema models were employed to evaluate the anti‐inflammatory potential, whereas antidiabetic activity was assessed using in vitro α‐amylase inhibition and in vivo oral glucose tolerance test (OGTT). Molecular docking and ADME/T analysis were employed to further analyze bioactive compounds identified using gas chromatography–mass spectrometry (GC–MS).

**Result:**

Antioxidant activity demonstrated a minimum inhibitory concentration (IC_50_) of 107.44 μg/mL for DPPH and 118.23 μg/mL for ABTS. In vitro anti‐inflammatory assays indicated a suppression of protein denaturation at a concentration of 102 μg/mL (IC_50_), where AECA (400 mg/kg) resulted in a 27% reduction in paw edema at 6 h in the mouse model. In vitro antidiabetic test indicated α‐amylase inhibition with an IC_50_ value of 70.72 μg/mL, and in the OGTT, a significant lowering of blood glucose was recorded at 120 min in mice. Strong binding affinities were observed for stigmasta‐5,24(28)‐dien‐3‐ol, identified using GC–MS, with values of −9.9 kcal/mol for α‐amylase and − 8.0 kcal/mol for cyclooxygenase‐2.

**Conclusion:**

*C. aerea* serves as an effective natural remedy for oxidative stress, inflammation, and hyperglycemia. These findings advocate for further clinical and mechanistic investigations to optimize therapeutic efficacy.

## Graphical abstract

This study revealed the antioxidant, anti‐inflammatory, and antidiabetic potentials of the seaweed *Chaetomortpha aerea* using in vitro, in vivo, and in silico approaches along with phytochemical analysis using gas chromatography–mass spectrometry. ADME/T analysis was also carried out to confirm drug likeness and toxicity prediction.

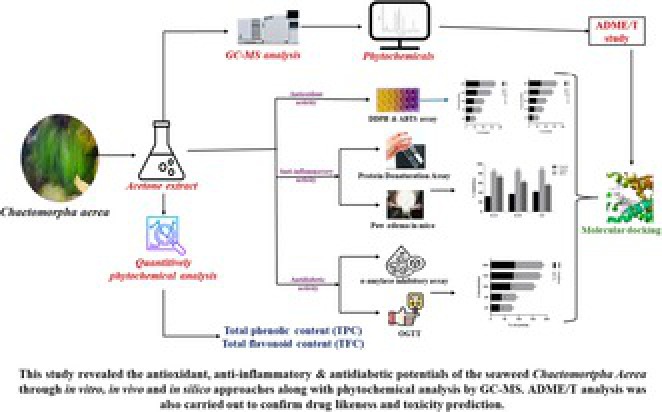

## 1 INTRODUCTION

Seaweed, also known as marine macro‐algae, has been an indispensable part of the human diet and a traditional medicine of coastal populations because of its nutritional, phytochemical, and therapeutic benefits.^1^ These algae are a source of proteins, vitamins, minerals, and polysaccharides, making them a suitable substitute for diets that are high in nutrients but low in calories.^2^ Seaweed has been used medicinally because it possesses bioactive compounds like sulfated polysaccharides, flavonoids, polyphenols, and various vitamins.^3^ Its antibacterial, antidiabetic, antioxidant, and anticancer properties have been the subject of numerous investigations.^4^, ^5^, ^6^

Reactive oxygen species are typically synthesized during a living organism's metabolism and are scavenged by both enzymatic and nonenzymatic defensive mechanisms. Stressful situations, however, can cause the molecular defense to malfunction and result in unsteadiness and extremely reactive free radicals, which can permanently harm physiological molecules like proteins, lipids, amino acids, and DNA.^7^ Therefore, oxidative stress has been linked to several illnesses, including neurological conditions, cancer, diabetes, hypertension, atherosclerosis, and inflammatory diseases.^7^, ^8^ Inflammation, a fundamental biological response triggered by injury and infection, is characterized by redness, swelling, heat, and discomfort. Even though chronic inflammation is essential for tissue healing, it is the underlying cause of many diseases and significantly affects morbidity and mortality globally.^9^ The Global Burden study estimates that inflammatory disorders have a significant impact, contributing to millions of deaths and disability‐adjusted life years (DALYs) non‐steroidal anti‐inflammatory drugs per year.^10^ Diabetes mellitus (DM), a class of metabolic diseases characterized by an irregular increase in blood glucose levels, is characterized by an imbalance in the production of insulin or insensitivity to the hormone's effects on the transmission of cellular receptor signals. There are two primary forms of DM: type 1 and type 2. About 90% of all cases of diabetes are type 2, which is defined by variable levels of insulin resistance and/or inadequate insulin production in cells.^11^

Manufactured antioxidants, including butylated hydroxytoluene, butylated hydroxyanisole, and butyl hydroxyquinone, as well as artificial antimicrobials, like sodium nitrite, sodium benzoate, and sorbic acid, are harmful and have the potential to cause cancer.^12^ Therefore, natural antioxidants are preferred by consumers over synthetic antioxidants. Current therapies for inflammation include corticosteroids and non‐steroidal anti‐inflammatory drugs (NSAIDs); although they reduce symptoms, they can cause organ damage, immunosuppression, and gastrointestinal problems.^13^ Pharmacological approaches are commonly used as part of current therapies for various illnesses. Insulin and oral hypoglycemic medications are widely used to treat type‐2 DM even though they can result in hypoglycemia, weight gain, insulin allergy, and gastrointestinal problems.^14^

The green alga *Chaetomorpha aerea* is found naturally in temperate and coastal regions, mostly in shallow water and intertidal areas. It forms dense mats on a variety of surfaces, including rocks.^15^ It has cylindrical filaments that are morphologically unbranched and have elongated cells, which range in hue from bright green to dark.^16^ Its diverse reserve of constituents confers upon it both nutritional and therapeutic properties. *C. aerea* is a vital component of the diet because of its rich protein, vitamin, and mineral content, which includes iodine, calcium, iron, and vitamins A and C.^17^ Its polyphenols, flavonoids, and sulfated polysaccharides have been demonstrated to possess significant antibacterial, antioxidant, and anti‐inflammatory properties in medical applications.^18^, ^19^, ^20^ Moreover, bioactive substances that can increase insulin production or improve sensitivity to the impact of insulin on cellular receptor signal transduction are the source of antidiabetic properties.^11^ These features highlight its potential as a medicine and demand more pharmacological investigation. Another in vitro study demonstrates the chloroform extract of *C. aerea's* antidiabetic properties by successfully blocking α‐amylase.^21^ Moreover, *C. aerea's* methanolic extract has antioxidant activity in vitro.^18^

However, there are limited comprehensive studies that integrate in vitro, in vivo, and in silico approaches to validate the multitarget therapeutic potential of *C. aerea* against oxidative stress, inflammation, and diabetes, despite the previously identified bioactivity. This investigation addresses that gap by conducting a comprehensive assessment of the bioactivity, phytochemical profile, and computational drug likeness of *C. aerea* to determine its translational significance.

## 2 MATERIALS AND METHODS

### 2.1 Chemicals

Methanol, Tween‐80, diazepam, and imipramine were used for analysis. Methanol and Tween‐80 were procured from Sigma‐Aldrich (St. Louis, MO, USA), whereas diazepam and imipramine were obtained from Square Pharmaceuticals PLC. All chemicals and reagents used were of analytical grade to ensure the accuracy and reliability of the experimental outcomes.

### 2.2 Collection and preparation of extracts

The marine green alga *C. aerea* was collected from Kutubdia, Cox's Bazar, Bangladesh (identified under accession number CU/DP/2023/03); acetone extract was obtained by drying the alga at room temperature, grinding, soaking, filtering (*Whatman filter paper*: grade *540*), and solvent evaporation using a rotary evaporator.

### 2.3 Quantitative phytochemical analysis

#### 2.3.1 Total phenolic content and total flavonoid content

To verify the presence of total phenolic content (TPC) of acetone extract of *C. aerea* (AECA), the absorbance of a standard (gallic acid) at 765 nm was measured using a Folin–Ciocalteu. For quercetin equivalents, the total flavonoid content (TFC) was evaluated using AlCl_3_ and CH_3_CO_2_K, with absorbance at 415 nm.^22^

#### 2.3.2 Gas chromatography–mass spectrometry analysis

AECA was analyzed using a Shimadzu TQ 8040 mass spectrometer equipped with electron ionization, coupled to a Shimadzu GC‐17A gas chromatograph integrated with an Rxi‐5‐ms capillary column. Helium was used as the carrier gas at a flow rate of 0.6 mL/min. Gas chromatography–mass spectrometry (GC–MS) interface temperature was maintained at 280°C, and the scan range was set between 40 and 350 amu. Compound identification was performed by comparing the obtained spectra with the NIST GC–MS library (version 08‐S).

### 2.4 Experimental animal

Albino mice (4–5 weeks old, 20–25 g) were obtained from BCSIR (Chittagong) and acclimated for 1 week before experimentation. They were housed under controlled conditions (25 ± 2°C, 45%–55% relative humidity, 12‐h light–dark cycle) in the Department of Pharmacy, University of Chittagong, Bangladesh. Sterile polypropylene cages, clean water, and a standard diet were provided, with food deprivation for 12 h before and during the experiment.

### 2.5 Animal killing

The study was approved by the Ethical Review Board, University of Chittagong, Faculty of Biological Sciences (AERB‐FBSCU‐2025107‐02), and conducted following the 2013 Animal Euthanasia criteria and the Swiss Academy of Sciences criteria.

### 2.6 Acute oral toxicity test

Three groups of six Swiss albino mice (20–25 g) were used in the acute toxicity test, which was conducted following OECD Guideline 423. ACEA (200 and 400 mg/kg) was administered to the test groups, whereas distilled water was given to controls. Serious toxicity was observed in the first trials at 1000 and 4000 mg/kg, which led to dose reduction. Mice were evaluated every day for 14 days, with body weight, food, and water intake recorded, and their toxic level was monitored for 24 h. Following the AVMA Guidelines (2020), mice were administered intraperitoneal pentobarbital (200 mg/kg) at the end of the study, and their organs were histologically examined.^23^, ^24^

### 2.7 Determination of antioxidant activity

#### 2.7.1 DPPH free radical scavenging activity

The 2,2‐diphenyl‐1‐picrylhydrazyl (DPPH) assay, following Sundaram et al., evaluated the scavenging activity of ACEA; 0.004% DPPH was added to dilutions (25–200 μg/mL), and the mixture was incubated for 30 min. Absorbance at 517 nm was measured using UV spectrophotometry to calculate radical scavenging activity^25^:

$$\left(\%\right)\;\text{Free radical scavenging}=\left[\left(A_{0}–A_{1}\right)/A_{0}\right]\times 100.$$

where *A*_0_ represents the control's absorbance and *A*_1_ the extract's absorbance.

#### 2.7.2 ABTS free radical scavenging method

Following Sundaram et al., the 2,2′‐azino‐bis(3‐ethylbenzothiazoline‐6‐sulfonic acid (ABTS) assay was used to measure the antioxidant activity of *C. aerea* extract. ABTS• + radical cation was obtained by combining 7 mM ABTS with 2.45 mM K_2_S_2_O_8_ and incubating the mixture in the dark for 24–48 h. The extract (1:10 in acetone) was mixed with ABTS•+, and absorbance at 734 nm was recorded at 0, 5, and 10 min to calculate the inhibition percentage^25^:

$$\;I\%=\frac{A_{t=0}-A_{t}}{A_{t=0}}\times 100.$$

where *I* represents the percentage inhibition of ABTS, *A*_*t*=0_ the control sample's absorbance (*t* = 0 h), and *A*_*t*_ the tested sample's absorbance at 5 or 10 min.

### 2.8 Determination of anti‐inflammatory activity

#### 2.8.1 In vitro protein denaturation assay

With a few adjustments, the experiment was conducted using the methodology stated by Joshi et al.^26^ Five sets of test solutions comprising 125, 62.5, and 31.25 μg/mL of aspirin (standard) or AECA were prepared (per concentration), and each tube was filled with 5 mL of albumin 25%. The solutions were then gently mixed and left to remain at room temperature for 15 min. The reaction mixture was kept at 70°C in a water bath for 10 min to induce denaturation. After cooling, turbidity was measured using a spectrophotometer adjusted to 660 nm. The percentage inhibition of denaturation was calculated using the following formula:

$$\%\text{Inhibition of denaturation}=100\times \left(1–A_{2}/A_{1}\right).$$

where *A*_1_ represents the control's absorbance and *A*_2_ the extract's absorbance.

#### 2.8.2 Carrageenan‐induced paw edema test

The carrageenan‐induced paw edema method, as described by Szekalska et al., was employed to evaluate anti‐inflammatory activity.^27^ Thirty mice were divided into five groups of six: group I (negative control) received 10 mL/kg of 1% Tween‐80, group II received carrageenan only, group III received 100 mg/kg of diclofenac sodium, and groups IV and V received 200 and 400 mg/kg of leaf extract, respectively. One hour after carrageenan injection (100 μL of 1% w/v in saline) into the right hind paw, treatments were administered orally. Paw circumference was measured at 0, 1, 2, 3, 4, 5, and 6 h postinjection to assess edema.

### 2.9 Determination of antidiabetic activity

#### 2.9.1 In vitro α‐amylase inhibitory assay

Antidiabetic activity was assessed using the α‐amylase inhibition test.^28^ After sample solutions (0.25–5000 μg/mL) were prepared in phosphate buffer (pH 6.9), they were incubated at 37°C with α‐amylase and starch. After the reaction with iodine and HCl was stopped, absorbance was measured at 630 nm to calculate the minimum inhibitory concentration (IC₅₀) using linear regression. The following equation was used to calculate the inhibitory activity of α‐amylase as a percentage of inhibition:

$$\%\text{Inhibition}=\frac{\left(\text{Absorbance control}-\text{Absorbance sample}\right)}{\text{Absorbance control}}\times 100.$$

#### 2.9.2 Oral glucose tolerance test

The study followed the methodology described by Small et al. to assess the antidiabetic potential of *C. aerea*.^29^ An oral glucose tolerance test (OGTT) was carried out on mice that had fasted overnight. Four groups were randomly selected from among the mice, irrespective of their sex. Six mice were included in each group. The treatments administered were as follows: 2% Tween for the negative control group, glibenclamide for the positive control group, and extracts for the test groups. The mice were given an oral glucose solution containing 2 g/kg 30 min after the corresponding treatments were administered. The mouse tails were then aseptically used to draw blood samples at 0, 30, 60, and 120 min after the glucose was administered.

### 2.10 In silico studies

#### 2.10.1 Molecular docking study

##### 2.10.1.1 Ligand preparation

Twelve minor metabolites were identified in the acetone extract from the seaweed *C. aerea* using GC–MS analysis. These molecules were retrieved in three‐dimensional (3D) SDF format for docking experiments from the PubChem database. The two‐dimensional (2D) SDF files were downloaded and converted into the 3D SDF format^30^ using Open Babel software in cases where the 3D SDF format was not available. Before docking simulations^31^ all ligands were energy minimized and converted into .pdbqt format using AutoDock Tools (version 1.5.6).

##### 2.10.1.2 Protein preparation

The structures of human cytochrome P450 CYP2C9 (PDB ID: 1OG5), cyclooxygenase‐2 (PDB ID: 5IKR), and pancreatic α‐amylase (PDB ID: 4 W93) were retrieved in PDB format from the RCSB Protein Data Bank (https://www.rcsb.org/structure) for antioxidant, anti‐inflammatory, and antidiabetic analyses. Water molecules and heteroatoms were removed using Discovery Studio 2020, and the proteins were then subjected to energy minimization using Swiss‐PdbViewer (version 4.1.0) via the conjugate gradient and steepest descent methods. The minimized PDB files were converted to .pdbqt format using AutoDock Tools (version 1.5.6) for further analysis.^32^

#### 2.10.2 Molecular docking analysis

Selected proteins were docked with *C. aerea* ligands using PyRx AutoDock Vina 1.2.0, which includes Python bindings, an extended force field, and new docking algorithms. A semiflexible approach was applied, treating the protein as rigid and the ligands as flexible. Active sites were defined using the co‐crystallized ligand, which was redocked to validate the protocol, with root mean square deviation values <2 Å considered acceptable. After validation, grid boxes (25 × 25 × 25 Å) were set at the co‐crystallized ligand positions. Docking interactions were visualized and analyzed using BIOVIA Discovery Studio Visualiser 2020.^33^

#### 2.10.3 ADME/T evaluation

The Absorption, Distribution, Metabolism, Excretion, and Toxicity (ADME/T) properties of ACEA's bioactive chemicals were assessed using the “rule of five” of Lipinski via SwissADME and pkCSM to assess drug likeness and pharmacokinetics.^34^

#### 2.10.4 Prediction of activity spectra for substances

The PASS (prediction of activity spectra for substances) online server was used to determine the biological activity of AECA compounds, predicting probable activity (Pa) and inactivity (Pi) for drug‐like molecules, with SMILES format conversion via PubChem.^35^

### 2.11 Statistical analysis

The data were represented as mean ± standard error of the mean (SEM). Dunnett's T‐test was utilized to establish statistical significance (*p < 0.05, **p < 0.01, and ***p < 0.001) of the control group. GraphPad Prism (version 8.0) was used for constructing the graph, and SPSS (version 25) was utilized for data analysis. Triplicate measurements were used in in vitro investigation.^36^

## 3 RESULTS

### 3.1 Quantitative phytochemical analysis

#### 3.1.1 Total phenolic and flavonoid concentration

TPC (22.93 ± 1.05 mg Gallic Acid Equivalent per gram (GAE/g) extract) was greater than the total TFC (17.28 ± 0.70 mg Quercetin Equivalent per gram (QUE/g) extract), as shown in Table 1.

**TABLE 1:** Total phenolic and flavonoid content in AECA.

Extract/standard	Total phenolic content (mg GAE/g extract)	Total flavonoid content (mg QUE/g extract)	IC_50_ value (μg/mL)
AECA	22.93 ± 1.05	17.28 ± 0.70	107.44
Ascorbic acid	–	–	72.62

Abbreviations: AECA, acetone extract of *Chaetomorpha aerea*; IC_50_, minimum inhibitory concentration.

#### 3.1.2 GC–MS analysis of *C. aerea*

**FIGURE 1:**
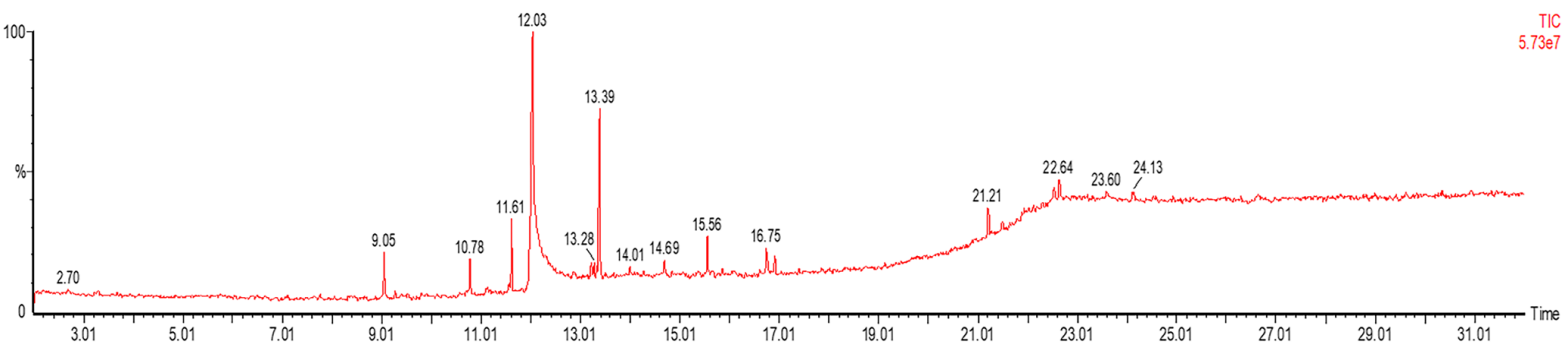
GC–MS (gas chromatography–mass spectrometry) chromatogram of acetone extract of *Chaetomorpha aerea* (AECA). GC–MS analysis of the AECA revealed 12 bioactive compounds of several classes, including plant sterols, fatty acid derivatives, and hydrocarbons.

A total of 12 metabolites were eluted between 2.5 and 32 min retention time from the AECA sample (Figure 1). Based on the NIST GC–MS library version 08‐S, these metabolites were identified (Table 2).

**TABLE 2:** GC–MS‐identified metabolites with their characteristics.

Serial number	Retention time (min)	Compound name	Concentration (%)
1	22.64	Stigmasta‐5,24(28)‐dien‐3‐ol	0.88
2	21.212	Cholesterol	0.79
3	16.915	Bis(2‐ethylhexyl)pthalate	3.7
4	15.561	9‐Octadecenamide	5.42
5	14.692	3‐Methyl‐2(2‐oxopropyl)furan	0.83
6	13.388	Phytol	16.93
7	12.047	*N*‐Hexadecanoic acid	52.17
8	11.612	Tetradecanoic acid,10,13‐dime	8.91
9	9.277	Hentriacontane	1.07
10	9.054	8‐Heptadecene	2.51
11	7.762	Fumaric acid	1.5
12	2.074	Thiophene,2,5‐di(benzoylthio)	0.97

Abbreviation: GC–MS, gas chromatography–mass spectrometry.

### 3.2 In vitro antioxidant activity

#### 3.2.1 DPPH radical scavenging activity

**FIGURE 2:**
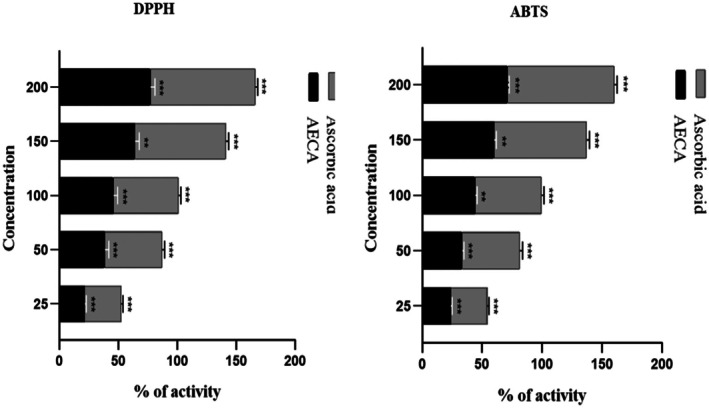
Percentage of scavenging activity of AECA (acetone extract of *C. aerea*) and ascorbic acid in DPPH and ABTS assays. DPPH and ABTS free radical scavenging activities of *Chaetomorpha aerea* acetone extract (AECA) at different concentrations. Values represent percentage inhibition (mean ± SEM [standard error of the mean]) with IC_50_ (minimum inhibitory concentration) values of 107.44 μg/mL (DPPH) and 118.23 μg/mL (ABTS) compared to ascorbic acid. Significance at *p < 0.05, **p < 0.01, and ***p < 0.001.

AECA exhibited a potent ability to scavenge free radicals, with an IC_50_ value of 107.44 μg/mL (Figure 2).

#### 3.2.2 ABTS radical scavenging activity

Similar to DPPH, AECA demonstrated strong radical scavenging activity in the ABTS assay, with an IC_50_ value of 118.23 μg/mL (Figure 2).

### 3.3 Determination of the anti‐inflammatory activity

#### 3.3.1 In vitro protein denaturation assay

**FIGURE 3:**
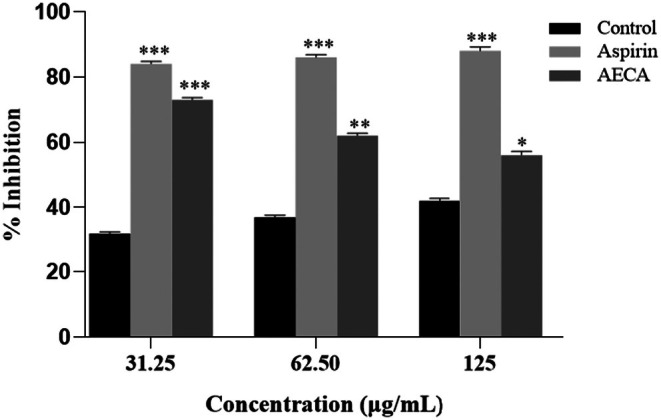
Anti‐inflammatory effect of AECA (acetone extract of *C. aerea*) by protein denaturation assay. Protein denaturation inhibition activities of AECA at different concentrations. Values represent percentage inhibition (mean ± SEM [standard error of the mean]) with IC_50_ (minimum inhibitory concentration) values of 102 μg/mL compared to aspirin. Significance at *p < 0.05, **p < 0.01, and ***p < 0.001.

AECA exhibited the highest percentage of protein denaturation inhibition at a concentration of 31.25 μg/mL (Figure 3). Having an IC_50_ value of 102 μg/mL, the AECA exhibited a considerable anti‐inflammatory impact compared to the negative control.

#### 3.3.2 Carrageenan‐induced paw edema test

Findings of in vivo anti‐inflammatory activity presented in Table 3 suggest that the paw thickness of the Carrageenan control group gradually increased in every hourly interval (1–6 h) after an abrupt increase at 5 h. At the fifth and sixth hours, the AECA at both dosages (200 and 400 mg/kg) significantly reduced paw thickness, whereas the 200‐mg/kg dose exhibited considerable activity against edematous response during the second to fourth hour. Strong activity is demonstrated by the 400‐mg/kg dose from the second to the sixth hour, nearly similar to the standard drug.

**TABLE 3:** In vivo anti‐inflammatory activity of AECA.

Treatment	Increase in paw edema thickness (mm), mean ± SEM						
	0 h	1 h	2 h	3 h	4 h	5 h	6 h
Control	3.45 ± 0.009	3.50 ± 0.014	3.51 ± 0.010	3.48 ± 0.08	3.44 ± 0.006	3.40 ± 0.06	3.39 ± 0.005
Carrageenan	3.43 ± 0.015	3.56 ± 0.013	3.58 ± 0.013	3.61 ± 0.09	3.65 ± 0.014	3.68 ± 0.08	3.61 ± 0.007
Diclofenac Na	3.40 ± 0.010**	3.33 ± 0.008***	3.28 ± 0.008***	3.25 ± 0.013***	3.23 ± 0.013***	3.21 ± 0.008***	3.20 ± 0.006***
AECA 200	3.43 ± 0.014	3.45 ± 0.006*	3.41 ± 0.017**	3.38 ± 0.026**	3.35 ± 0.017**	3.31 ± 0.009***	3.29 ± 0.010***
AECA 400	3.41 ± 0.019	3.40 ± 0.007**	3.36 ± 0.014***	3.32 ± 0.007***	3.28 ± 0.006***	3.26 ± 0.011***	3.25 ± 0.009***

*Note*: Significance at *p < 0.05, **p < 0.01, and ***p < 0.001.

Abbreviations: AECA, acetone extract of *Chaetomorpha aerea*; SEM, standard error of the mean.

### 3.4 Determination of antidiabetic activity

#### 3.4.1 In vitro α‐amylase inhibitory assay

**FIGURE 4:**
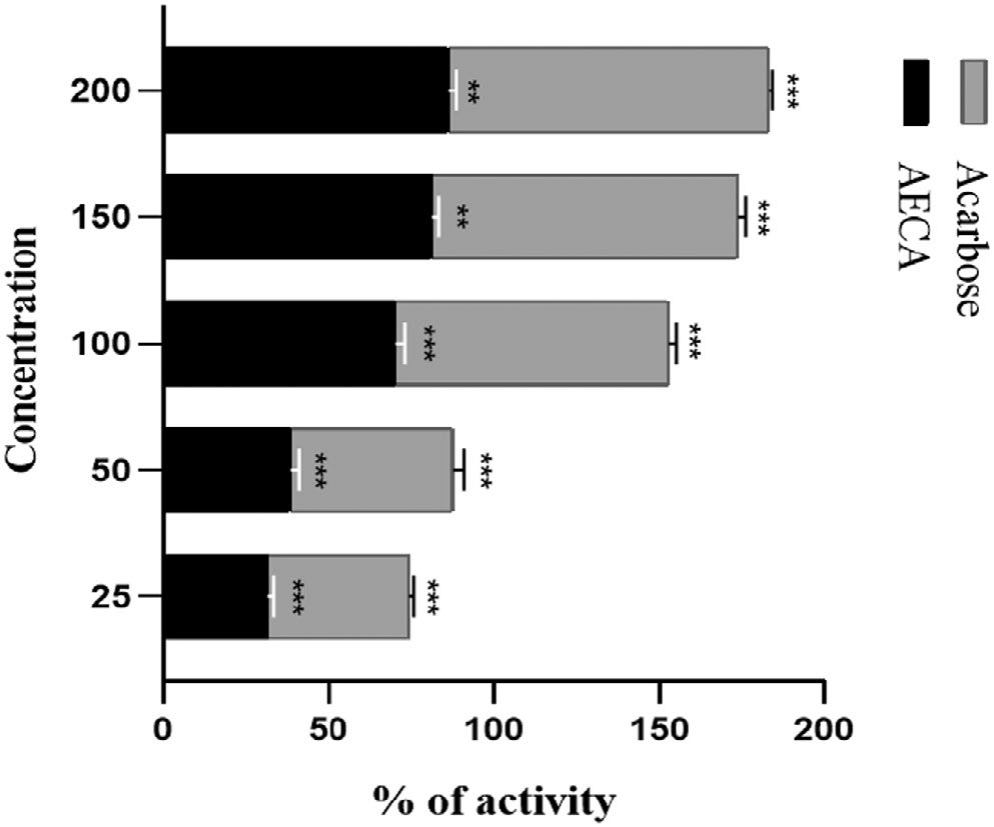
Percentage of inhibition of α‐amylase. α‐Amylase inhibition activity of *Chaetomorpha aerea* acetone extract (AECA) at different concentrations. Values represent percentage inhibition (mean ± SEM [standard error of the mean]) with IC_50_ (minimum inhibitory concentration) values of 70.72 μg/mL compared to acarbose. Significance at *p < 0.05, **p < 0.01, and ***p < 0.001.

Both AECA and standard acarbose used in this assay exhibited significant dose‐dependent inhibitory activity (Figure 4). The IC_50_ value for AECA and acarbose were 70.72 and 36.26 μg/mL, respectively.

#### 3.4.2 Oral glucose tolerance test

The result of the OGTT using AECA is presented in Table 4. The extract exhibited mild activity at 30 min at 400 mg/kg and significant activity after 60 and 120 min at 200 mg/kg. The strongest activity was observed after 120 min, exhibited by the extract at 400 mg/kg when the blood glucose level reduced to 5.6 from 10.1 mmol/L. The reference drug glibenclamide reduced the blood glucose level to 7.5, 6.1, and 3.9 mmol/L after 30, 60, and 120 min, respectively, of 2 g of oral glucose administration.

**TABLE 4:** Hypoglycemic activity of AECA.

Dose (mg/kg)	Blood glucose level (mmol/L)			
	0 min	30 min	60 min	120 min
Control	11.1 ± 0.78	10.4 ± 0.49	8.9 ± 0.26	7.1 ± 0.17
Glibenclamide	9.6 ± 0.38	7.5 ± 0.54*	6.1 ± 0.29*	3.9 ± 0.12**
AECA 200	10.3 ± 0.36	8.8 ± 0.32	7.2 ± 0.26**	5.6 ± 0.31**
AECA 400	10.1 ± 0.95	8.1 ± 0.20*	6.9 ± 0.20**	5.1 ± 0.15***

*Note*: Significance at *p < 0.05, **p < 0.01, and ***p < 0.001.

Abbreviation: AECA, acetone extract of *Chaetomorpha aerea*.

### 3.5 In silico study

#### 3.5.1 Molecular docking study

Detected compounds of AECA were docked against three major proteins, namely human cytochrome P450 CYP2C9 (PDB ID: 1OG5), human cyclooxygenase‐2 (PDB ID: 5IKR), and human pancreatic α‐amylase (PDB ID: 4 W93), for antioxidant activity, anti‐inflammatory activity, and α‐amylase inhibitory activity, respectively. Docking scores against these proteins are presented in Table 5.

**TABLE 5:** Docking score of the identified compounds of AECA against selected proteins.

PubChem ID	Compound name	Binding energy (kcal/mol)		
		4 W93	4 W93	5IKR
131 750 945	Stigmasta‐5,24(28)‐dien‐3‐ol	−9.9	−10	−8
569 794	Thiophene,2,5‐di(benzoylthio)	−7.7	−8.9	−7.1
8343	8‐Heptadecene	−6.1	−7.3	−6.3
5 280 435	Phytol	−6	−6.1	−4.9
5997	Cholesterol	−5.8	−6	−7.9
5 283 387	9‐Octadecenamide, (*z*)‐	−5.3	−5.5	−6.1
985	*N*‐Hexadecanoic acid	−5.3	−5.4	−5.3
12 410	Hentriacontane	−5.2	−6	−4.7
5 364 555	Bis(2‐ethylhexyl)phthalate	−5	−5.5	−5
11 005	Tetradecanoic acid,10,13‐dime	−5	−5.5	−5.9
545 772	3‐Methyl‐2(2‐oxopropyl)furan	−4.8	−5.2	−5.4
444 972	Fumaric acid,2‐chlorophenyle	−4.1	−5.3	−4.7
41 774	Acarbose	−7.1	–	–
54 670 067	Ascorbic acid	–	−5.1	–
51 081	Pefloxacin	–	–	–
5 388 992	Vincristine sulfate	–	–	–
2244	Aspirin	–	–	−6.5
3033	Diclofenac Na	–	–	–
2082	Albendazole	–	–	–

Abbreviation: AECA, acetone extract of *Chaetomorpha aerea*.

##### 3.5.1.1 Molecular docking related to antioxidant activity

**FIGURE 5:**
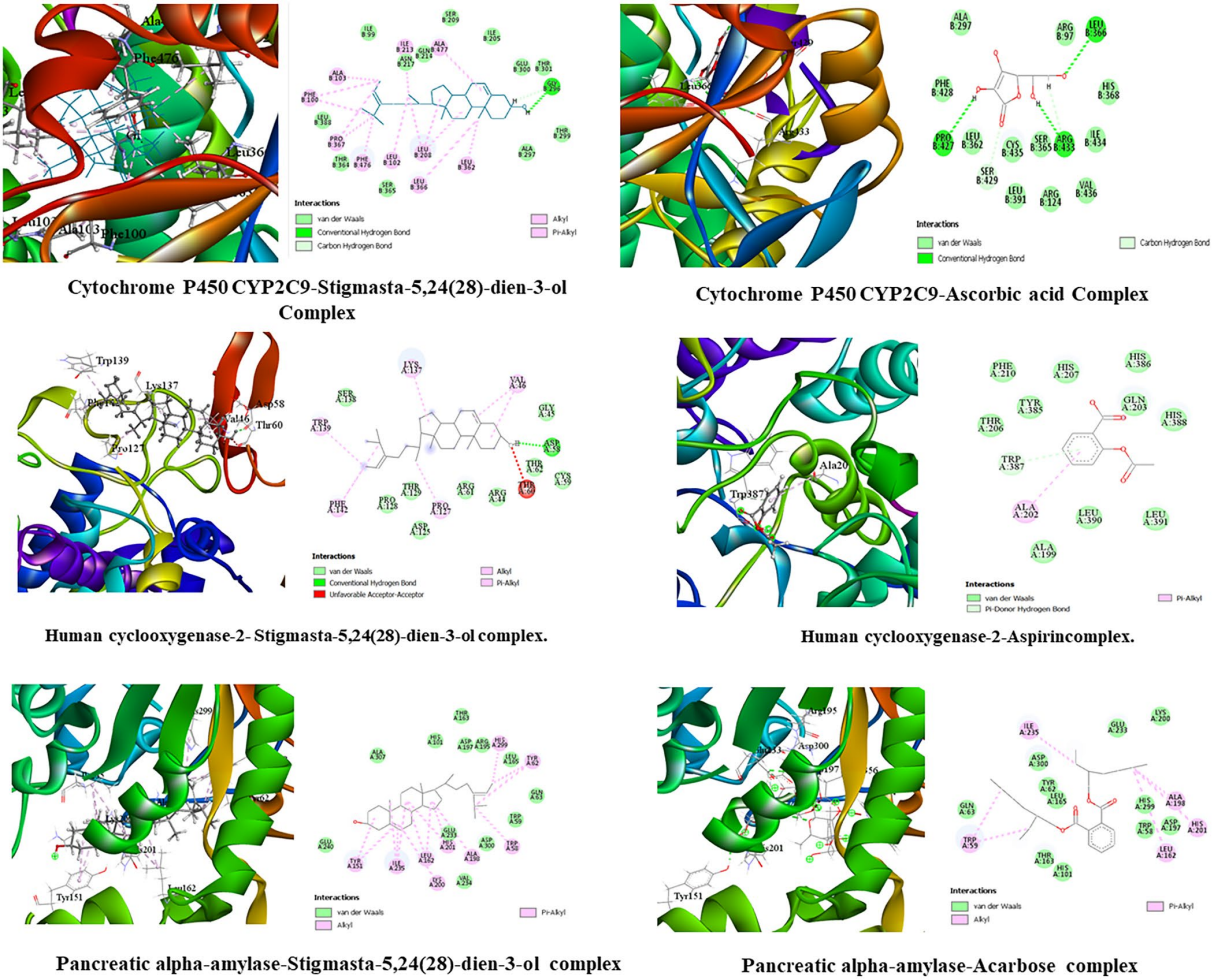
Molecular docking interactions. Visual representation of human cytochrome P450 CYP2C9 (PDB ID: 1OG5), human cyclooxygenase‐2 (PDB ID: 5IKR), and human pancreatic α‐amylase (PDB ID: 4 W93) with top docked compound and standard, respectively.

Antioxidant activity demonstrated in molecular docking, along with bond types and bond length, is presented in Table 6, and the top docked compounds and the standard ascorbic acid are shown in Figure 5.

**TABLE 6:** List of bond types and amino acids involved in the top compound and standard with respective proteins.

Protein	PubChem ID	Compound name	Binding energy (kcal/mol)	Hydrogen bond		Hydrophobic bond		
				Conventional	Carbon–hydrogen	Pi‐alkyl	Alkyl	Others
**4 W93**	131 750 945	Stigmasta‐5,24(28)‐dien‐3‐ol	−9.9			TRP58, TYR62	LEU162, ALA198, LYS200, ILE235	Pi‐Sigma: TYR62
						TYR151		
						HIS201, HIS299		
	41 774	Acarbose	−7.1	TYR151, GLU233, HIS201	GLU233, ASP356, ASP300			
**1OG5**	131 750 945	Stigmasta‐5,24(28)‐dien‐3‐ol	−10	GLY296		PHE100, PHE476	ALA103, LEU208, ALA477, LEU36, LEU366, PRO367, ILE213, LEU102	Pi‐Sigma: PHE476
	54 670 067	Ascorbic acid	−5.1	LEU366, ARG433, PRO427	SER429			
**5IKR**	131 750 945	Stigmasta‐5,24(28)‐dien‐3‐ol	−8	ASP58		TRP139, PHE142	VAL46, LYS137, PRO127	
	2244	Aspirin	−6.5	TRP387		ALA202		

##### 3.5.1.2 Molecular docking related to anti‐inflammatory activity

The molecular docking activity linked to anti‐inflammatory action and information on bond lengths and types are presented in Table 6. Figure 5 shows the top docked compounds and standard aspirin against human cyclooxygenase‐2 (PDB ID: 5IKR) in both 2D and 3D.

##### 3.5.1.3 Molecular docking related to antidiabetic activity

Molecular docking activity related to antidiabetic activity and bond types and bond length are presented in Table 6, and the top docked compounds and standard acarbose are shown (both 2D and 3D) in Figure 5.

#### 3.5.2 ADME/T and PASS prediction properties

Lipinski's five‐point scale assessed the drug likeness of *C. aerea* compounds, confirming compliance with the “rule of five” for oral bioavailability. ADME/T characterization of the compounds is presented in Table 7. PASS analysis revealed that the compounds provided strong antioxidant, antidiabetic, and antibacterial potential with higher Pa values than Pi (Table 8).

**TABLE 7:** ADME/T analysis of AECA compounds.

Name of compounds	Absorption		Distribution		Metabolism	Excretion	Toxicity		Drug likeliness	Bioavailability
	Water solubility (log mol/L)	Intestinal absorption (% absorbed)	VDss (human) (log L/kg)	BBB permeability (log BB)	CYP3A4 substrate	Total clearance (log mL/min/kg)	AMES toxicity	Hepatotoxicity		
Thiophene,2,5‐di(benzoylthio)	−6.22	92.677	0.01	0.059	Yes	−0.041	No	No	Yes	0.55
Fumaric acid,2‐chlorophenyl	−0.642	71.771	−1.026	−0.127	No	0.89	No	No	Yes	0.85
8‐Heptadecene	−8.277	91.208	0.644	0.948	Yes	1.929	No	No	Yes	0.55
Hentriacontane	−6.092	85.891	−0.016	1.222	Yes	2.188	No	No	Yes	0.55
Tetradecanoic acid,10,13‐dime	−4.952	92.691	−0.578	−0.027	No	1.693	No	No	Yes	0.85
*N*‐Hexadecanoic acid	−5.562	92.004	−0.543	−0.111	Yes	1.763	No	No	Yes	0.85
Phytol	−7.554	90.71	0.468	0.806	Yes	1.686	No	No	Yes	0.55
3‐Methyl‐2(2‐oxopropyl) furan	−1.023	97.089	−0.06	0.055	No	0.668	No	No	Yes	0.55
9‐Octadecenamide, (*z*)‐	−7.074	90.218	0.281	−0.389	Yes	1.959	No	No	Yes	0.55
Bis(2‐ethylhexyl)phthalate	−6.47	92.45	0.36	−0.175	Yes	1.898	No	No	Yes	0.55
Cholesterol	−6.917	93.723	0.382	0.763	Yes	0.589	No	No	Yes	0.55
Stigmasta‐5,24(28)‐dien‐3‐ol	−6.715	94.642	0.179	0.764	Yes	0.619	No	No	Yes	0.55

Abbreviation: AECA, acetone extract of *Chaetomorpha aerea*; AMES, Salmonella/microsome mutagenicity assay; VDss, Volume of distribution; BBB, Blood brain barrier.

**TABLE 8:** PASS prediction value of compounds from *Chaetomorpha aerea* seaweed for antidiabetic, antioxidant, cytotoxic, anti‐inflammatory, antiarthritic, and anthelmintic activities.

Compound name	Antioxidant		Anti‐inflammatory		Antidiabetic	
	Pa	Pi	Pa	Pi	Pa	Pi
Stigmasta‐5,24(28)‐dien‐3‐ol	0.196	0.057	0.575	0.037	0.257	0.207
Cholesterol	0.198	0.056	0.572	0.038	0.405	0.061
Thiophene,2,5‐di(benzoylthio)	0.074	0.036	–	–	0.396	0.013
Bis(2‐ethylhexyl)phthalate	0.134	0.122	0.537	0.046	0.229	0.048
Phytol	0.475	0.008	0.458	0.07	0.365	0.083
Hentriacontane	0.17	0.079	0.424	0.084	0.622	0.012
Tetradecanoic acid,10,13‐dime	0.222	0.045	0.515	0.052	0.323	0.07
*N*‐Hexadecanoic acid	0.222	0.045	0.515	0.052	0.323	0.07
8‐Heptadecene	0.281	0.027	0.622	0.027	0.336	0.024
9‐Octadecenamide	0.167	0.082	0.384	0.104	0.233	0.091
3‐Methyl‐2(2‐oxopropyl)furan	0.145	0.06	0.572	0.038	0.278	0.035
Fumaric acid	0.411	0.011	0.602	0.031	0.512	0.021

Abbreviation: PASS, prediction of activity spectra for substances.

## 4 DISCUSSION

*C. aerea*, a filamentous green seaweed, is characterized by tubular branched structures that mainly grow in maritime conditions.^37^ Packed with phytochemicals, including polysaccharides, phenolic compounds, and fatty acids, it exhibits a wide range of pharmacological properties, making it a good source for medicinal applications.^38^ Our research therefore attempts to assess this green filamentous seaweed's potential for hypoglycemic, antioxidant, cytotoxic, anti‐inflammatory, antarthritic, and anthelmintic activities.

TPC and TFC were estimated utilizing the regression equation for gallic acid (*y* = −0.0074*x* + 21.708, *R* ^2^ = 0.051) and quercetin (*y* = −0.0074*x* + 21.708, *R* ^2^ = 0.051), respectively. Table 1 suggests that the TPC (20.93 ± 1.05 mg GAE/g extract) was higher compared to the TFC (15.78 ± 0.70 mg QUE/g extract). GC–MS analysis identified several potential bioactive metabolites. *N*‐Hexadecanoic acid, a principal constituent, is documented to possess antioxidant effects and may influence inflammatory pathways.^39^ Phytol exhibits considerable antioxidant, anti‐inflammatory, and antidiabetic properties by affecting lipid metabolism and glucose regulation.^40^ 9‐Octadecenamide, a compound linked to fatty acid amides, may indirectly promote metabolic health, whereas direct evidence is limited. Derivatives of tetradecanoic acid may provide anti‐inflammatory benefits.^41^

A strong free radical scavenging activity was demonstrated by AECA where the IC_50_ value was 107.44 μg/mL with the regression equation *y* = 0.3001*x* + 17.758, *R* ^2^ = 0.9721, whereas the IC_50_ value of standard ascorbic acid was 72.62 with the regression equation *y* = 0.3172*x* + 26.965, *R* ^2^ = 0.967. Together with ascorbic acid, AECA's antioxidant activity significantly increased in a dose‐dependent manner. The peak scavenging effect was found at 200 μg/mL of AECA, which was less than that of ascorbic acid but still comparable (Figure 2). Like DPPH, the ABTS assay exhibited a potent scavenging activity of AECA. The IC_50_ value of AECA was 118.23 μg/mL with the regression equation *y* = 0.2719*x* + 17.854, *R* ^2^ = 0.9952, whereas standard ascorbic acid revealed an IC_50_ value of 108.72 μg/mL with the regression equation *y* = 0.281*x* + 19.454, *R* ^2^ = 0.9857. The scavenging activity increased significantly with dose, where the highest activity was found at 200 μg/mL of AECA, which was further less than that of ascorbic acid but still comparable (Figure 2). Influenced by N‐hexadecanoic acid (52.17%), which may stabilize lipid peroxidation, and phytol (16.93%), a diterpene alcohol with free radical scavenging properties, the extract exhibits promising antioxidant potential.^39^ These compounds, along with natural algal antioxidants, illustrate the mechanisms in spirulina (*Arthrospira platensis*), renowned for its phycocyanin‐mediated antioxidant properties.^42^

The protein denaturation assay is performed to assess the anti‐inflammatory action of a compound by evaluating its ability to prevent protein denaturation, a major process in inflammation.^26^ The protein denaturation assay demonstrated that AECA exhibited the highest inhibition at 31.25 μg/mL, indicating strong anti‐inflammatory potential (Figure 3). The IC_50_ value of 102 μg/mL suggests moderate efficacy, with a significant impact compared to the control. However, AECA was less effective than aspirin across all concentrations. The anti‐inflammatory action of AECA was also evidenced in the carrageenan‐induced paw edema in mice. The 400‐mg/kg dose significantly decreased edema compared to the standard (Table 3). Derivatives of tetradecanoic acid (8.91%), fatty acid amide, and 9‐octadecenamide (5.42%) could aid in lowering pro‐inflammatory mediators (e.g., cyclooxygenase‐2, nuclear factor kappa B).^43^ Comparable mechanisms are elucidated in *Chondrus crispus* (Irish moss), where sulfated polysaccharides inhibit the production of tumor necrosis factor‐α and interleukin‐6.^44^

AECA demonstrated potential as an α‐amylase inhibitor in our investigation, as evidenced by its IC_50_ value of 70.72 μg/mL, whereas the standard acarbose exhibited an IC_50_ value of 36.26 μg/mL. Both AECA and standard acarbose used in this assay exhibited significant dose‐dependent inhibitory activity (Figure 4). Also in OGTT, there was a significant reduction in the level of blood glucose in mice in the AECA group, which demonstrated the antihypertensive effect of the alga (Table 4). Phytol's role in lipid metabolism and glucose regulation aligns with *Ecklonia cava*, a brown alga whose phlorotannins enhance insulin sensitivity by inhibiting α‐glucosidase.^45^ Similar to *Ulva lactuca*, the fatty acids present in *C. aerea* may influence peroxisome proliferator‐activated receptor gamma (PPAR‐γ) Influenced by N‐hexadecanoic acid (52.17%), which may stabilize lipid peroxidation, and phytol (16.93%), a diterpene alcohol with free radical scavenging properties, the extract exhibits promising antioxidant potential pathways, therefore improving glycemic regulation in preclinical animal models.^46^

Molecular docking study of the AECA compounds against the respective proteins demonstrated more binding energy compared to standard drugs (Table 2). Moreover, the bond types and the amino acids engaged in bond construction are presented in Table 6. Also, Figure 5 clearly shows that the interactions of the top compounds at the protein's active location further strengthened the in vitro findings.

A five‐point scale was used by Lipinski to evaluate the compounds of *C. aerea*. To evaluate a small molecule's potential for drug likeness and identify whether a chemical with a certain therapeutic action has properties, Ro5 is a theoretically and computationally potent method. Ro5 states that when (i) log *p* > 5, (ii) hydrogen bond donors >5, (iii) hydrogen bond acceptors >10, and (iv) the molecular weight >500, low oral bioavailability may occur. Our analysis shows that every molecule possesses drug‐like characteristics and complies with the five rules of Lipinski (Table 4).^47^

Compounds from *C. aerea* were tested for their antioxidant, antidiabetic, and antibacterial qualities using the PASS online tool.^35^ The powerful compounds exhibited a Pa value that was higher than that of Pi (Table 5).

## 5 CONCLUSION

The findings of this study demonstrate that the ACEA seaweed may be a potent source of antidiabetic and antioxidant properties and a moderate source of antibacterial versus gram‐negative *Escherichia coli* and *Salmonella typhi* bacteria. A moderate cytotoxic effect was also evident and demonstrated by this study. Furthermore, using the Lipinski rule of five, molecular docking simulation revealed that a number of bioactive candidate compounds possessed drug‐like properties and a high binding affinity with specific proteins. Additionally, the experimental outcomes for the seaweed components agree with the PASS predictions. To support the present findings, more investigations have to be conducted to shed light on the mechanisms of the relevant bioactive phytoconstituents.

## AUTHOR CONTRIBUTIONS

**Md. Mahmudul Hasan:** Conceptualization; data curation; formal analysis; visualization; writing – original draft; writing – review and editing. **Md. Abdul Alim:** Investigation; writing – original draft; writing – review and editing. **Md. Safayat Hossen Momen:** Investigation; writing – original draft; writing – review and editing. **Md. Shahidul Islam:** Writing – original draft; writing – review and editing. **Sajjad Hossen Chowdhury:** Writing – original draft; writing – review and editing. **Mohammad Rashed:** Writing – original draft; writing – review and editing. **Fahmina Hoque:** Writing – original draft; writing – review and editing. **S. M. Moazzem Hossen:** Conceptualization; methodology; project administration; supervision; writing – original draft; writing – review and editing.

## FUNDING INFORMATION

The authors received no specific funding for this work.

## CONFLICT OF INTEREST STATEMENT

The authors have no known competing interests.

## ETHICS STATEMENT

The approval of this study was granted by the Ethical Review Board. Departmental ethical consent number AERB‐FBSCU‐20250107(2).
